# Bridging Political Divides: Perceived Threat and Uncertainty Avoidance Help Explain the Relationship Between Political Ideology and Immigrant Attitudes Within Diverse Intergroup Contexts

**DOI:** 10.3389/fpsyg.2019.01236

**Published:** 2019-06-14

**Authors:** Brandon D. Stewart, Fyqa Gulzaib, David S. M. Morris

**Affiliations:** School of Psychology, University of Birmingham, Birmingham, United Kingdom

**Keywords:** uncertainty, threat, political ideology, intergroup, bias

## Abstract

The political divide between liberals and conservatives has become quite large and stable, and there appear to be many reasons for disagreements on a wide range of issues. The current research sought to explain these divides and to extend the Uncertainty-Threat Model to intergroup relations, which predicts that more dispositional, perceived-threat and uncertainty-avoidance will be related to more political conservatism. Given that conservatism is also often related to more negativity to low-status groups such as immigrants, the relationship between political ideology and negative attitudes toward immigrants may be mediated by more threat and uncertainty-avoidance. Study 1 tested this mediational hypothesis in a correlational design and showed that both uncertainty-avoidance and perceived realistic and symbolic threat significantly mediated the relationship between political ideology and attitudes toward immigrants, and that perceived threat was the more influential mediator. Study 2 extended threat management to perceived threats from unspecified outgroups, as opposed to the immigrant outgroup, and it replicated all significant mediations. Study 3 replicated the mediations observed in Studies 1 and 2 for political ideology to attitudes toward immigrants with uncertainty-avoidance and perceived threat from immigrants as mediators; it further replicated the mediations to the negative attitudes measure that had been used in Study 2 and it extended it to an objective and indirect bias measure [i.e., Affect Misattribution Procedure (AMP)]. Overall, almost all of the results supported the idea that perceived threat and uncertainty-avoidance both mediate the relationship between political ideology and attitudes toward immigrants, and that threat management, as opposed to negativity bias, may be a central concern separating liberals and conservatives. Within all three studies, we also observed more evidence for the Uncertainty-Threat Model predictions than we did for the alternative Extremity Hypothesis, which predicted a quadratic relationship between political ideology and threat and uncertainty, and between political ideology and attitudes toward immigrants.

## Introduction

The divisions between liberals and conservatives have become quite large and stable, and are potentially expanding (Koleva et al., 2012; Hibbing et al., 2014; Jost et al., 2017). Overall, these political groups tend to disagree on a wide range of issues that often appear to not have a common underlying motivation (e.g., same-sex marriage, taxes, flag burning, animal testing, immigration, etc.). To complicate matters, there are a number of factors that have been proposed to explain the differences between these groups including differences in social dominance orientation (SDO), differences in negativity bias, and differences in endorsement of moral foundations (Graham et al., 2011; Ho et al., 2012; Hibbing et al., 2014). We believe that an essential difference on these divides revolves around threat perceptions and uncertainty-avoidance.

One promising theoretical model proposes critical differences that relate to threat and uncertainty, which can shed light on these political divides, and may help to bridge them and improve dialogue between ideological groups (Jost et al., 2003, 2017; Jost and Napier, 2012). In their Uncertainty-Threat Model research, Jost et al. (2007, 2017), Jost and Napier (2012) provide some evidence that liberals and conservatives differ in their attention to and management of threats (i.e., Threat Management related to perceived terrorism, belief in a dangerous world, or death anxiety), and in the avoidance of uncertainty (i.e., Uncertainty-Avoidance), where more uncertainty avoidance and more attention to threats were associated with less liberalism and more conservatism (Jost et al., 2007). Here we use Jost’s broad “Threat Management” terminology, which encompasses many different types of threat (i.e., threat to self-esteem, mortality salience, ideological threat to the system, etc.); we, however, sought to extend research in this area and to test a specific type of threat – perceived threat toward one’s group, which is a fundamental motive within intergroup relations based upon Intergroup Threat Theory (ITT) (Kurzban et al., 2001; Stephan et al., 2009, 2015), and which may be included within the broader threat management classification that Jost uses. We conceptually replicated some effects from the Uncertainty-Threat Model and we extended them to the context of immigration and extended them by using a standard measure of perceived realistic and symbolic threat based upon ITT (Stephan et al., 1999).

### Testing the Uncertainty-Threat Model

The Uncertainty-Threat Model (UTM) may provide theoretical insights into observed differences between liberals and conservatives on a variety of issues, including negative responses to low status groups such as immigrants. In Jost and colleagues’ original review of political orientation research, they argued that conservatives’ resistance to change and resistance to equality is motivated by higher needs to manage threat and uncertainty (Jost et al., 2003); these needs can be due to either *dispositional* motives (e.g., personality), or a conservative shift in needs could occur from *temporarily* activated motives (i.e., priming) that temporarily shifts and strengthens affiliation with threat and/or uncertainty avoidance. Jost et al. (2007) provided some of the first evidence to explicitly test these ideas. In their paper, they observed that uncertainty-avoidance (e.g., need for order, intolerance of ambiguity, and lack of openness) and threat management (e.g., death anxiety, system threat, and/or perceptions of a dangerous world) each predicted more conservatism. They, however, did not support the alternative model proposed by the worldview validation perspective (Greenberg and Jonas, 2003) that predicted that more uncertainty-avoidance and threat management should be related to more extreme political ideology, on both the left and the right (Jost et al., 2007). Thus, there is some support for the Uncertainty-Threat Model that argues that “both *temporary, situational* factors and *chronic, dispositional* tendencies pertaining to the avoidance of uncertainty and the management of threat are therefore hypothesized to affect ideological preferences” (Jost et al., 2007, p. 990); these chronic tendencies are proposed to be related to more conservatism and less liberalism (Jost et al., 2007; Jost, 2017).

While the Uncertainty-Threat model could be insightful, there is debate about whether and how uncertainty and threat relate to political ideology and how it might relate to intergroup relations. Our primary goal in this research was to seek to explain this political divide. Therefore, we tested the ability of measured/dispositional uncertainty-avoidance and perceived-threat to mediate the relationship between political ideology and attitudes toward immigrants that are highly relevant to current political debates within a diversifying world; these debates are likely to continue for decades to come as countries grapple with the need for immigration and this need is likely to continue because economic growth is currently tied to increased efficiency and increased population growth, which will depend upon immigration because the current US birth rate is below population replacement levels. In our research, we tested the hypotheses that more conservatism and less liberalism is related to more negative attitudes toward immigrants (Hypothesis 1a), that more conservatism would be related to more dispositional uncertainty-avoidance and more perceived threat (Hypothesis 1b), and that higher uncertainty-avoidance and perceived threat would mediate the relationship between political ideology and attitudes toward immigrants (Hypothesis 1c). At this early stage, we did not focus on the temporary conservative shift hypothesis and instead sought to test the chronic dispositional hypothesis.

The finding of liberals showing less avoidance of uncertainty and less of a focus on threats and conservatives showing more of each could be increased when encountering intergroup situations. Research has shown that thinking about or encountering intergroup situations can increase uncertainty and perceived threat (Grieve and Hogg, 1999; Stephan et al., 2009). Thus, in intergroup situations of non-immigrants thinking about immigrants, a motivation of uncertainty-avoidance and threat-management should be predictive of responses to outgroups that are emphasized by the intergroup context (Jost et al., 2003, 2017). There is, however, little research on threat and uncertainty together, and less on the relationship between political ideology, threat, uncertainty, and immigrants together. Thus, we next examine these separate areas of research.

Some researchers argue that uncertainty can increase deliberation or compromise in negotiations and reduce confidence (Marcus et al., 2000; MacKuen et al., 2010). This position, would then argue that increased uncertainty should also lead to less biased responding. Yet, increased uncertainty has been demonstrated to be related to increased intergroup bias in both minimal group paradigms and with real-world groups (Grieve and Hogg, 1999; Federico et al., 2013) and to be related to increased confidence in one’s attitudes and social identity, which can lead to more attitude bias (Hogg and Mullin, 1999; Van den Bos et al., 2005; McGregor, 2006). Thus, more reliance on avoiding uncertainty could be related to more attitude bias.

Previous research, however, has also demonstrated that conservatives are more influenced by uncertainty in situations in which threats have been highlighted (Haas, 2016). In her research, Haas measured support for compromise on the Affordable Care Act (i.e., Obamacare), and manipulated uncertainty and threat by asking participants to imagine either a home invasion scenario (threat) in which the culprit was either inside the house (certain) or trying to get in (uncertain). In the low threat control condition, participants imagined someone arriving at home during the day and ringing the doorbell; the person was either someone they knew (certain) or did not know (uncertain; Haas and Cunningham, 2014). This research observed that conservatives expressed less support for compromise with liberals, but only when uncertainty was paired with threat (Haas, 2016). This research would indicate that conditions that paired threat and uncertainty may increase bias for conservatives.

In regard to threat, it is clearer that attention to threat can often lead to a variety of biases at the intergroup level. In Riek et al. (2006) using 95 samples, perceived threat was shown to have a large influence on increased negative intergroup bias (average *r* = 0.43). Numerous other studies have also indicated that perceiving more threats are linked with increased intergroup bias and increased negative attitudes toward immigrants (Zarate et al., 2004; Pereira et al., 2010; Butz and Yogeeswaran, 2011). In regard to political orientation, research has demonstrated that liberals show less acceptance of inequality (Duckitt, 2001; Jost et al., 2003; Duckitt and Sibley, 2009), have more positive attitudes toward gays and lesbians, Muslim Americans, and Arabs (Whitley and Lee, 2000; Cunningham et al., 2004; Webster et al., 2014), show less outgroup hostility (Kugler et al., 2014), and have more positive feelings toward non-normative groups such as gays, lesbians, atheists, and Muslims, who are seen as deviating from Judeo-Christian values (Luguri et al., 2012). In addition, Jost et al. (2017), in their extensive meta-analysis, reviewed 174 tests of the hypothesized connection between feelings of insecurity and threat and more conservatism or authoritarianism and they found a positive relationship between conservatism and more subjective threat (weighted average, *r* = 0.26). Reviews have also supported the hypothesis that conservatives are more likely and liberals less likely to have a lower tolerance for uncertainty; the review notes that, over nine studies, there was a moderate to large association between conservatism and lower tolerance for uncertainty (weighted average *r* = −0.33; Jost, 2017). Thus, liberals are likely to show less negative attitudes toward low-status outgroups such as immigrants, and responsiveness to threat is likely to increase these negative attitudes, and potentially explain this relationship. This evidence and the evidence showing that uncertainty-avoidance is often related to more negative attitudes and that conservatives tend to show more uncertainty-avoidance, lead us to predict that conservatives would show more negative attitudes toward immigrants than would liberals (Hypothesis 1a). Based upon Jost’s model and because conservatives are proposed to show more threat-avoidance and more uncertainty-avoidance, we predicted that both of the *dispositional* variables of Perceived Threat and Uncertainty-Avoidance would mediate the relationship to Attitudes toward immigrants (Hypothesis 1c).

A secondary goal of the current research was to test the Uncertainty-Threat Model against the Extremity Hypothesis. Several researchers argue that needs to reduce uncertainty and threat should lead to endorsement of any extreme ideology (i.e., extremity hypothesis) regardless of one’s beliefs and political ideology (McGregor et al., 2001; Greenberg and Jonas, 2003; Hogg, 2012). Thus, needs to reduce threat and uncertainty should be higher at both ends of the political range. One standard way to measure this is to test the linear and quadratic regression effects between political ideology and threat and uncertainty-avoidance (Jost and Napier, 2012; van Prooijen et al., 2015). A quadratic effect would show that as political ideology became more liberal or more conservative, the relationship to threat and uncertainty-avoidance would be stronger (i.e., a u-shaped relationship). A linear only trend would support the Uncertainty-Threat Mode because it would show that more conservatism and less liberalism was related to more threat and uncertainty avoidance in a linear fashion.

Little research evidence, however, has examined the extremity hypothesis (i.e., extreme responding on both sides of the political spectrum), and the research that does exist is somewhat ambiguous (Jost and Napier, 2012). Jost et al. (2003) found that 7 of 13 studies in their meta-analysis observed only a linear relationship between uncertainty and conservatism (i.e., support for the uncertainty-threat model), while 6 studies showed both a linear relationship and a quadratic relationship (i.e., support for uncertainty-threat model and for extreme responding on both ends of the political spectrum). Thus, according to Jost and Napier (2012), p. 91 “psychological needs to reduce uncertainty and threat are associated with political conservatism in particular and not ideological extremity in general.” In newer research, van Prooijen et al. (2015) have observed that people on the left and right political extremes showed more derogation of immigrants and more derogation of societal groups in general than did political moderates, however, their research tested attitudes to multiple societal groups simultaneously, instead of individually, which could have changed participants’ responses compared to having multiple participants rate only a single group; such question order context effects have been demonstrated previously in which the order of questions frames the questions that follow; thus, the order of questions regarding multiple societal groups could affect the interpretation and response to those groups (Schwarz et al., 1990; Schuldt et al., 2015); future research will need to investigate this phenomena more thoroughly. Regardless, van Prooijen et al., did observe a small (Cohen et al., 2003) quadratic relationship to derogation of immigrants by liberals and conservatives on the extreme ends of the spectrum (*R*^2^ = 0.01), and they showed that there was a descriptively larger linear relationship in which there was more derogation by conservatives and less by liberals (*R*^2^ = 0.17). Overall, there is some evidence showing that people at the political extremes show more extreme responding, but the research also demonstrates more extreme responses by conservatives on negative responses to societal groups in general and toward immigrants.

Jost et al. (2007) and Jost and Napier (2012) have, however, provided additional evidence of the relationship between conservatism, and uncertainty and threat reduction. In their original work, Jost et al. (2007) showed that political conservatism was related to more uncertainty-avoidance and more threat, but these factors were not significantly related to ideological extremism noted in the extremity hypothesis; this pattern was demonstrated reliably in three different studies conducted in three different geographic regions within the US. In our current research, we sought to replicate these relationships between political ideology and more threat and more uncertainty-avoidance, and to extend them to attitudes toward immigrants. In Hypothesis 2a, we predicted that conservatives would show more perceived threat and more uncertainty-avoidance than liberals, and that we would observe a linear relationship only (Uncertainty-Threat Model) and not a quadratic relationship as suggested by the worldview validation perspective (Greenberg and Jonas, 2003; Hogg, 2012). While this prediction is contrary to Burke and colleagues’ meta-analytic findings (Burke et al., 2013), Burke’s analysis focused on mortality threats, which were not measured in our research; we, instead, focused on perceived threats to one’s ingroup that were not related to physical death in the measure of Perceived Threat that we used; this measure is based upon ITT (Stephan et al., 1999), and while threat of death or physical harm could have been included in a measure of perceived realistic and symbolic threat, Stephan and colleagues, to date, have not included it within their standard measure. As a result, we continued the use of this standard measure of perceived realistic and symbolic threat used in ITT by Stephan and colleagues.

We also predicted that liberals would show less negative attitudes toward immigrants and conservatives would show more negative attitudes because existing evidence has demonstrated this relationship (Luguri et al., 2012; Kugler et al., 2014; Brooks et al., 2016). Thus, if the extremity hypothesis from the worldview validation perspective were to be supported, a chronic tendency toward more threat and uncertainty-avoidance should be related to participants being more polarized in their view of immigrants as their chronic tendency to avoid threat/uncertainty was greater (e.g., very liberal participants could be more positive and very conservative participants could be more negative, or both could be more negative). Thus, the quadratic relationship test could be applied to attitudes toward immigrants in addition to threat and uncertainty-avoidance. Given that liberals were predicted to be less negative and conservatives more negative toward immigrants, we also predicted that there would be linear relationship between political ideology and attitudes toward immigrants in which more conservatism was related to more negative attitudes, but that the quadratic term would be non-significant (i.e., a natural extension of the extremity hypothesis in which one’s initial preference would be increased or be more negative; Hypothesis 2b).

A recent review by Crawford (2017), has indicated that the way threat has been defined within the motivated social cognition (Jost et al., 2003) and negativity bias (Hibbing) perspectives is too broad. Crawford argues that threats can be differentiated into *meaning threats* and into *physical threats*. The *meaning threats* “include more abstract concerns regarding the violation of one’s sense of belonging, identity, purpose, significance, continuity, or certainty” while *physical threats* “include more concrete concerns regarding the violation of one’s physical safety and well-being through the potential of death or other physical trauma” (Crawford, p. 356). His review argues that while liberals and conservatives are differentially influenced by *physical* threats, they are, similarly, influenced by *meaning* threats. Within our current set of studies, we have not focused on *physical threats* (i.e., threats of death or bodily harm); instead, we have used the ITT perspective (Stephan et al., 2015) that has not differentiated between realistic threats about competition for resources (e.g., taking jobs) and threats about physical safety (e.g., experiencing crimes or physical trauma); the ITT, instead, has focused on concerns for the ingroup’s existence, resources, quality of life, and values (Rios et al., 2018). Thus, we have used the standard ITT measure of perceived threat to one’s group resources and values that, to date, has not included threats of death, physical trauma, or experiencing crimes, at either the concrete level or at the conceptual level (Stephan et al., 1999, 2009). Theoretically, a measure of perceived realistic and symbolic threat could tap into the concrete concerns for physical threat, and future research could test other measures that incorporate this type of threat. Overall, the current research used a concept of threat that is more closely tied to *meaning threats* and also not in the realm of mortality salience; this research, however, is limited by using a pre-existing measure that did not set out to differentiate these types of threat.

### Research Overview

We sought to explain the divide between liberals and conservatives and sought to extend the Uncertainty-Threat model to intergroup relations regarding immigrants and in relation to perceived threats highlighted in the ITT. Given that liberals tend to show less negative attitudes toward low status and non-normative groups, we hypothesized that liberals would show less negative attitudes toward immigrants and conservatives would show more negative attitudes toward them. We next tested whether this relationship would be significantly mediated by both uncertainty-avoidance and perceived threat, and whether these factors were equally influential. In follow-up studies, we extended perceived threat from immigrants to include perceived threats from unspecified outgroups, and added a measure of negative attitudes and an implicit measure of attitudes toward immigrants.

## Study 1

### Hypotheses

Hypothesis 1a: Conservatives would express more negative attitudes toward immigrants than would liberals. We also, tentatively, predicted that liberalism would be related to higher rates of helping immigrants, though given the placement of this variable at the very end of the study, this hypothesis is exploratory.

Hypothesis 1b: More conservatism would be related to more Uncertainty-Avoidance and more Perceived-Threat.

Hypothesis 1c*:* Both *dispositional* factors of Perceived-Threat and Uncertainty-Avoidance would mediate the relationship of Political Ideology to Attitudes.

Hypothesis 2a: Conservatism would have a positive linear relationship with Uncertainty-Avoidance and Perceived-Threat, but the quadratic relationship would be non-significant.

Hypothesis 2b: Conservatism would have a linear relationship with Negative Attitudes, but the quadratic relationship would be non-significant.

### Methods

#### Participants

Two hundred and six participants from the United States were recruited from the online platform Prolific.ac and they completed the online study for monetary compensation. Within the Prolific.ac platform, we recruited only participants who indicated that their nationality was US and that they were residing in the US; thus, no participant was an immigrant. Participants were between 18 and 65 years old (*M* = 31.00, *SD* = 11.35) with 77.2% White, 25.7% Conservative, and 64.1% male participants. We recruited roughly 200 participants in order to have.8 power for 2 predictors in the model with a nearly medium effect size (*d* = 0.46), which is based upon effect sizes observed for the smallest factor in previous research (Jost et al., 2007; Jost and Napier, 2012). See Supplementary Materials for Supplementary Data Sheet 1 (i.e., Data Dictionary for Study 1) and Supplementary Data Sheet 2 (i.e., Data File for Study 1).

#### Materials

##### Filler task 1

The first filler task was composed of four items from the Need for Cognition scale (Cacioppo and Petty, 1984) that were not related to political ideology (*α* = 0.75, “Need for Cogition4” to Political Ideology, *r* = −0.007). Examples of the items included: “I like tasks that require little thought once I’ve learned them” and “I find satisfaction in deliberating hard for many hours.” The items were answered on a 1–5 response scale from Extremely Uncharacteristic to Extremely Characteristic, and after reverse scoring two items, higher scores indicated more Need for Cognition.

##### Perceived threat from immigrants

This standard scale from ITT (Stephan et al., 1999, 2015) consisted of fifteen items that measured threat levels based on seven symbolic threat and eight realistic threat items that were presented in a randomized order. The scale used a seven-point, vertical scale from (1) Disagree Strongly to (7) Agree Strongly. An example of the items included: “The values and beliefs of immigrants regarding social relations are not compatible with the beliefs and values of most Americans.” Because the subscales share a common theme of threats to the ingroup (Stephan et al., 1999) and were highly correlated (*r* = 0.75, *p* < 0.001, α = 0.94), we used all fifteen items in the index of perceived threat, which has been used in previous research (Tausch et al., 2007; Verkuyten, 2009; Tip et al., 2012; Schmid and Muldoon, 2015; Stephan et al., 2015). After reverse-scoring 8 items, scores were averaged and higher scores indicated more perceived threat (*M* = 3.40, *SD* = 1.24, α = 0.94).

##### Uncertainty-avoidance

We used Jost et al. (2007) Uncertainty-Avoidance measure. This measure was the mean of items from the 10 item Openness to Experience subscale of the Big Five Inventory (BFI; John et al., 2008, e.g., “I am someone who is original, and comes up with new ideas”) and from the same 4 item Intolerance for Ambiguity measure used by Jost (Budner, 1962, e.g., “People who insist upon a yes or no answer just don’t know how complicated things really are”). Both scales were rated from (1) Disagree strongly to (7) Agree strongly and the items were randomized within scales; after reverse scoring two items, scores were averaged and higher scores represented more uncertainty-avoidance (*M* = 3.10, *SD* = 0.83, α = 0.81).

##### Filler task 2

The second filler task was placed between the mediators and the outcome measure, and was composed of four additional items from the Need for Cognition scale (α = 0.86, “Need for Cogition2nd4” to Political Ideology, *r* = 0.047). Examples of the items included “I would prefer complex to simple problems,” and “I only think as hard as I have to.” After reverse scoring two items, scores were averaged and higher scores indicated more Need for Cognition.

##### Attitudes scale

a five-item Feeling Thermometer (Alwin, 1992; Saguy et al., 2009) was used as the Attitude measure. Participants rated their feelings toward immigrants on five opposite pairs of evaluative dimensions (Warm-Cold, Negative-Positive, Friendly-Unfriendly, Suspicious-Trustworthy, Admiration-Disgust) using a nine-point, vertical scale (e.g., the top, 1 = _______ to 9 = _______). After reverse-scoring two items, higher scores indicated more negative attitudes (*M* = 4.03, *SD* = 1.55, α = 0.95). A separate measure that was not incorporated into the attitude measure above was completed as a pilot test for future items for Study 2. Participants completed some pilot questions toward immigrants (Approval, Acceptance, Liking, Disdain, Hatred; adapted from Stephan et al., 2002) by rating “… the degree to which you feel _____ toward immigrants” from (0) No liking at all (9) Extreme liking, depending upon the adjective rated.

##### Secondary measures

See Supplementary Table 1 in Supplementary Materials for descriptions and analyses of these measures. The measures included the SDO scale, the Death Avoidance scale, the Fear of Death scale, and a System Threat item.

##### Additional questions on three separate pages

“In order to demonstrate that you are a real person, please complete some of these mathematics problems: 6 × 7 = ___; 8 + 5 = _____.” “Would you be willing to help out immigrants, who are living in poverty, by providing your email address to be contacted about helping out? __No, __Yes.” (No email address was collected). “In the US, left-wing ideas are often but not exclusively supported by the Democratic Party, and right-wing ideas are often but not exclusively supported by the Republican Party. We are interested in where you see yourself in the political spectrum from 1 = Very Right, to 5 = Neither, to 7 = Very Left” (*M* = 4.60, *SD* = 1.81).

#### Design

Political orientation was a measured predictor variable, while Perceived Threat and Uncertainty-Avoidance were mediator variables, and Attitudes toward Immigrants was the outcome variable. The two mediators of Perceived Threat (Stephan et al., 1999) and Uncertainty-Avoidance (Jost et al., 2007) were counterbalanced in order to methodologically control for order effects. The continuous, Political Orientation measure was followed by Perceived Threat and then Uncertainty-Avoidance in orders one (U-A: openness then ambiguity) and two (U-A: ambiguity then openness), or it was followed by Uncertainty-Avoidance and then Perceived Threat in orders three (U-A: openness then ambiguity) and four (U-A: ambiguity then openness). Participants were randomly assigned to one of these four, counterbalanced orders (see Supplementary Table 1 in Supplementary Materials for these four orders).

#### Procedure

Participants completed informed consent and then some demographic variables including gender, ethnicity, age, English as a second language, residence status, and political ideology. Participants rated their personal political orientation on a nine-point, vertical scale from (1) extremely conservative to (9) extremely liberal, with moderate as the mid-point (5) as was used in Jost et al., 2007. The political orientation item has often been included within demographics in previous research (Fitzgerald and Wickwire, 2012; Feinberg and Willer, 2013; Crawford et al., 2015; Janoff-Bulman and Carnes, 2016). The mean political orientation score was 5.65 (*SD* = 2.17).

Next, participants completed Filler Task 1 that included four filler questions from the Need for Cognition scale that were uncorrelated with political ideology (*r* = 0.007), and that provided a small separation from the next task. Participants were then randomly assigned to one of two orders of the mediators by being told to select the letter that appeared at the top of a list of letters. (Each list was randomly ordered for each participant; thus, participants were not self-selecting into an order. Whichever letter was chosen had been randomly ordered by the computer and participants did not know what each letter represented. Moreover, participants believed they were doing this to check that the system was recording their responses correctly). Participants then completed either the Perceived Threat scale first and then the Uncertainty-Avoidance scale, or they completed the opposite order. After completing these mediators, participants completed Filler Task 2 (four filler items from the Need for Cognition scale).

Participants next completed the five-item Feeling Thermometer (Alwin, 1992; Saguy et al., 2009) as the Attitude measure (*M* = 4.03, *SD* = 1.55, α = 0.95). They then completed five additional attitude items that were being pilot tested for a later study.

To further test some relationships suggested by the Uncertainty-Threat Model, we included some secondary measures of death avoidance, fear of death, and system threat that had been used by Jost et al. (2007). Participants first completed a Death Avoidance scale (5 items, α = 0.92, *M* = 3.73, *SD* = 1.68) and Fear of Death scale (*M* = 3.91, *SD* = 1.58, α = 0.91; Wong et al., 1994) to test the proposed positive relationship to conservatism. Participants also completed a single item of system threat (Jost et al., 2007) and then the SDO scale with sixteen items (*M* = 2.58, *SD* = 1.25, α = 0.95, Pratto et al., 1994) to test the positive relationships between political ideology and system threat, and between political ideology and social dominance. Next, participants completed some simple mathematics, filler questions and then a secondary, behavioral measure of helping that was being pilot tested. They answered a yes/no question about whether they would be willing to help immigrants, who are living in poverty, by providing their email address in order to be contacted about helping out; no email addresses were actually collected. Finally, participants completed questions about a left-right political orientation item and then questions about the purpose and how positive their day was, and then were debriefed. The purpose and positive day questions were filler questions.

### Results

Prior to testing the main hypotheses, we coded the four orders of the mediators and conducted a regression with Order, Political Orientation, and the interaction in the model for each outcome measure. We observed non-significant Order × Political interactions for Attitudes toward Immigrants and for Attitude Bias2 (pilot-tested items); for all analyses, we observed *p* > 0.146 and *R*^2^ < 0.011. A Linear regression was conducted next to investigate the hypothesis that Political Orientation would be associated with Attitudes toward immigrants. Higher scores reflected a more liberal political ideology. As hypothesized, Political Orientation was significantly and negatively related to negative Attitudes, *R*^2^ = 0.27, β = −0.52, *t* = -8.64, *p* < 0.001, with bootstrapped *b* = −0.37, 95%BCa CI [−0.45, −0.29], *p* < 0.001, which indicated that, within a narrow confidence interval, higher liberalism was associated with less negative Attitudes (Hypothesis 1a); all Confidence Intervals (CI) used Bias Corrected intervals (BCa CI) as recommended by Hayes (2013) regardless of whether they were called “BCa CI” or “CI”. Political Orientation was also significantly and negatively related to the second Attitude measure being pilot tested, *R*^2^ = 0.27, β = −0.51, *t* = -8.57, *p* < 0.001, with bootstrapped *b* = −0.42, 95%BCa CI [−0.51, −0.32], *p* < 0.001.

To test the extremity hypotheses, we centered Political Orientation and computed the quadratic term using the mean-centered variable based upon recommendations for quadratic regressions (Cohen et al., 2003; van Prooijen et al., 2015). We added the Political Orientation variable in Step 1 and the Quadratic variable in Step 2 of the regression model with Uncertainty-Avoidance and we observed a Quadratic *R*^2^ < 0.001, β = −0.01, *t* = −0.13, *p* = 0.894 with bootstrapped *b* < −0.01, 95%BCa CI [−0.02, 0.02]. This same analysis was performed for Perceived Threat and found a Quadratic *R*^2^ < 0.001, β = −0.03, *t* = −0.48, *p* = 0.631, bootstrapped *b* < −0.01, 95%BCa CI [−0.03, 0.02]. Both non-significant effects suggested that participants at the extreme ends of political ideology did not endorse threat or uncertainty-avoidance significantly more (Hypotheses 2a), which replicates the non-significant, quadratic effects observed by Jost et al. (2003, 2007). We also performed these analyses for the relationship between Political Ideology and Attitudes toward immigrants. The Quadratic term was non-significant, *R*^2^ < 0.001, β = −0.013, *t* = −0.20, *p* = 0.839 with bootstrapped *b* < −0.01, 95%BCa CI [−0.04, 0.03], suggesting that participants at either extreme end of the political spectrum were not responding with more negative attitudes toward immigrants (Hypothesis 2b). Finally, a binary logistic regression analysis was conducted to test whether Political Orientation would be positively associated with the binary, behavioral-variable of Helping Immigrants who are living in poverty. We observed that more liberalism was significantly related to more Helping of immigrants (Hypothesis 1a), β = 0.28, *SE* = 0.08, *Wald* = 10.97, *p* = 0.001, *Odds Ratio* = 1.32, 95%BCa CI [1.12, 1.55], Cox and Snell *R*^2^ = 0.06.

#### Mediation

**FIGURE 1:**
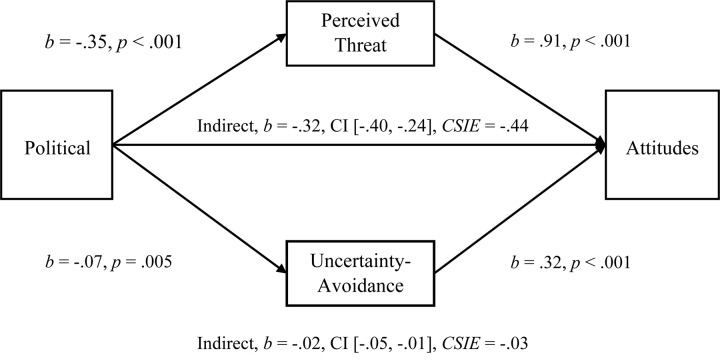
Simultaneous, multiple mediation of political orientation to attitudes toward immigrants with perceived threat and uncertainty-avoidance as mediators. Brackets represent 95% bias corrected confidence intervals from a 5000-sample bootstrap test.

We next tested the multiple-mediational hypothesis in which Perceived Threat and Uncertainty-Avoidance (*r* = 0.218) were proposed to significantly mediate the Political Orientation to Attitudes toward Immigrants relationship. We performed a simultaneous, multiple-mediation test using PROCESS analysis in SPSS with bias corrected confidence intervals to 5000 samples as recommended for multiple mediators by Hayes (2013). As hypothesized, both Perceived Threat and Uncertainty-Avoidance significantly mediated the Political Orientation to Attitudes relationship (see Figure 1; Hypothesis 1c). In evaluating the mediators, Threat was significantly larger with a comparison *b* = −0.29, CI [−0.38, −21] (see Supplementary Table 1 in Supplementary Materials for mediations using the Symbolic Threat subscale that replicate the effects observed with the entire Symbolic and Realistic Perceived Threat scale). We next tested the mediations for the relationship of Political Orientation to Pilot-tested Attitudes and observed that both Uncertainty-Avoidance (*b* = −0.02, CI [−0.05, −0.01], *CSIE* = −0.03) and Threat (*b* = −0.38, CI [−0.48, −0.29], *CSIE* = −0.47) were significant mediators. The mediational analyses for Behavioral Helping were also supportive of the model, but only for the Threat variable (see Supplementary Table 1 in Supplementary Materials).

Both mediators were also significant when entered into separate mediations (Uncertainty-Avoidance, *b* = −0.03, CI [−0.07, −0.01], *R*^2^ = 0.06, *CSIE* = −0.05, and Threat, *b* = −0.33, CI [−0.42, −0.26], *R*^2^ = 0.27, *CSIE* = −0.46). In addition, the Left-Right measure of Political Orientation produced the same pattern of results and levels of significance for all analyses. For both political variables, all patterns of results and levels of significance remained the same for all analyses in a sample of 181 participants after removing participants who failed to indicate that they were born in the United States in the second set of demographics questions in Study 1. Even though all 206 participants indicated that their Nationality was US in the prolific.ac screening criteria, some had failed to verify this in our demographic questions; we improved these questions in later studies.

### Discussion

Political Orientation was significantly and linearly associated with Perceived Threat and Uncertainty-Avoidance, but the quadratic effect, which tested the extremity hypothesis, was not significant; this pattern replicated the non-significant quadratic effects of Jost et al. (2003, 2007). In an extension of these predictions, the quadratic effect was also non-significant for the Political Orientation to Attitudes toward immigrants, which again shows a lack of support for the extremity hypothesis based upon the worldview validation perspective (Greenberg and Jonas, 2003; Hogg, 2012). Moreover, we observed that both Uncertainty-Avoidance and Perceived Threat were significant mediators in a multiple-mediation model of Political Orientation to Attitudes, and Perceived Threat was a significantly more influential mediator; these mediations were replicated with the Symbolic Threat subscale. Thus, the current research found support for the Uncertainty-Threat Model, and it extended this model to intergroup relations regarding immigrants and to Stephan et al.’s (1999) perceived threat scale.

## Study 2

Study 2 aimed to extend the measure of threat to *unspecified* outgroups and to test whether uncertainty-avoidance and perceived threat from unspecified outgroups, as opposed to immigrants, mediated the relationship between political orientation and attitudes toward immigrants. We adapted a perceived threat to specific groups scale (Stephan et al., 2002) to be about unspecified outgroups in much the same way that the SDO measure is about hierarchy in relation to unspecified groups. A second aim was to extend the measurement of attitudes to include only negative ratings in order to demonstrate that the effects occur on only negative attitudes and that they did not occur solely due to liberals responding more positively and conservatives responding on the neutral mid-point in Study 1. We hypothesized that political orientation would again be significantly and negatively correlated with attitudes and also with the new, negative attitude measure in addition to the helping immigrants variable. Second, we hypothesized that both uncertainty-avoidance and the perceived threat from unspecified outgroups would mediate the negative relationships between political ideology and attitudes toward immigrants, and between political ideology and negative attitudes, and in a replication of Study 1, threat would be a more influential mediator. Finally, we anticipated replicating the non-significant quadratic effects on the relationships between political ideology and the mediators and between political ideology and both measures of attitudes.

### Methods

#### Participants

A sample of three hundred and eight participants was recruited from Prolific.ac. All participants indicated within the Prolific.ac platform that they were born in the US and currently resided in the US; thus, no participant was considered an immigrant. They were between 18 and 74 years old (*M* = 31.53, *SD* = 12.12) with 80.5% White, 22.1% Conservative, and 50.6% male participants. We recruited roughly 300 participants in order to have 0.85 power for 2 predictors and 0.82 with 3 predictors in the model with a small effect size (*d* = 0.39) based upon Study 1 results estimated from the multiple-mediation model. See Supplementary Materials for Supplementary Data Sheet 3 (i.e., Data Dictionary for Study 2) and Supplementary Data Sheet 4 (i.e., Data File for Study 2).

### Design

The order of the measures and mediators was the same as in Study 1 with a few changes. The deviations from Study 1 included the following: (1) The ambiguity items and openness items were randomized within a single, Uncertainty-Avoidance questionnaire instead of within separate questionnaires, (2) Study 2 used only two orders because Uncertainty-Avoidance was now a single scale; thus, the Perceived Threat and Uncertainty-Avoidance mediators were again counterbalanced to control for order effects, (3) The Perceived Threat measure was changed from “threat from immigrants” to “threat from unspecified outgroups” using an adaptation of Stephan et al.’s (2002) measure with 24 items (see Supplementary Materials), and the single-item system threat question was included at the end of the perceived threat measure, (4) The death avoidance and threat from death scales were removed because of non-significant findings in Study 1, (5) an attitude scale with only negative adjectives was added after the first attitude scale from Study 1 in order to tap only negativity, and finally (6) seven filler items were added before the helping immigrants question. Participants were randomly assigned to one of the two, counterbalanced orders of the mediators.

#### New Materials

##### Perceived threat from unspecified outgroups

We used the 24-item Perceived Threat measure that we changed to be about (unspecified) outgroups by adapting Stephan et al.’s (2002) threat from ethnic minority groups measure. Within the instructions, participants were provided with this definition for outgroups: “In this section of the study, we will ask you to think about “OUTGROUPS.” For the purposes of this study, an Outgroup is any group or groups of which you DO NOT class yourself as being a member of, or belonging to, and that you do not identify with” (see Supplementary Table 1 in Supplementary Materials for the full measure). There were 12 symbolic threat items (e.g., “My group has very different values than outgroups.”) and 12 realistic threat items (e.g., Too much money is spent on educational programs that benefit outgroups). The measure used a seven-point, vertical scale from (1) Disagree Strongly to (7) Agree Strongly. After reverse scoring, we averaged the items and a higher score indicated more perceived threat from unspecified outgroups (α = 0.96).

##### Negative attitude scale

This scale included rating five negative feelings toward immigrants (Disapproval, Resentment, Dislike, Disdain, Hatred; adapted from Stephan et al., 2002). Participants were asked to “Please indicate the degree to which you feel _________ toward immigrants” on a nine-point, vertical scale from (0) No _________ to (9) Extreme *_________.* Higher scores represented more negative attitudes toward immigrants *(M* = 2.49, *SD* = 1.99, α = 0.95).

##### Filler task 3

A third filler task was placed between the attitude outcome measures and the final questions about mathematics, helping immigrants, and making errors within the study. It was composed of seven items from the Need for Cognition scale (α = 0.82).

#### Procedure

Following the rating of political orientation within the demographic questions, participants completed the same Need for Cognition Filler Task 1 as in Study 1 (α = 0.75). They then were randomly assigned to complete either the Perceived Threat from Unspecified Outgroups (24 items, α = 0.96, *r* = 0.87, *M* = 3.67, *SD* = 1.31) scale first and then the Uncertainty-Avoidance scale (*M* = 2.95, *SD* = 0.80, α = 0.81), or they completed the opposite order as was done in Study 1. Next, participants completed the same need for cognition Filler Task 2 as from Study 1 (α = 0.81).

Participants then completed the first Attitudes scale, which was the same as in Study 1 and included participants rating their feelings toward immigrants on five opposite pairs of evaluative dimensions (*M* = 3.87, *SD* = 1.66, α = 0.94). They then completed the Negative Attitude scale, which included rating five negative feelings toward immigrants (Disapproval, Resentment, Dislike, Disdain, Hatred (*M* = 2.49, *SD* = 1.99, α = 0.95). Participants next completed the SDO scale used in Study 1 (*M* = 2.36, *SD* = 1.18, α = 0.95). After completing Filler Task 3 (7 items from Need for Cognition scale; α = 0.82) and two mathematics items, participants completed the same yes/no helping-immigrants behavioral measure from Study 1, and then were debriefed.

### Results

We coded the two orders of the mediators and conducted a regression with Order, Political Orientation, and the interaction in the model for each outcome measure. We found non-significant Order x Political interactions for the Attitudes measure and the Negative Attitude measure; all interaction *p* > 0.713 and *R*^2^ < 0.001. Two linear regressions were performed to test the expected negative relationships between Political Orientation and Attitudes toward Immigrants and between Political Orientation and Negative Attitudes toward Immigrants. As expected, Political Orientation was significantly and negatively related to more negative Attitudes (Hypothesis 1a), *R*^2^ = 0.29, β = −0.54, *t* = −11.09, *p* < 0.001, with bootstrapped *b* = −0.43, 95%BCa CI [−0.51, −0.35], *p* < 0.001, and it also was significantly and negatively related to more negativity on the Negative Attitude measure (Hypothesis 1a), *R*^2^ = 0.19, β = −0.43, *t* = -8.38, *p* < 0.001, with bootstrapped *b* = −0.41, 95%BCa CI [−0.52, −0.31], *p* < 0.001; Because higher scores on Political Orientation were related to a more liberal ideology, the results indicated that higher liberalism was associated with less negative attitudes. Moreover, we created an Averaged Attitude score for Attitudes and Negative Attitudes and then standardized it; Political Orientation was significantly and negatively related to this standardized-Averaged Attitude score, *R*^2^ = 0.27, β = −0.51, *t* = −10.50, *p* < 0.001 (see the Supplementary Table 1 in Supplementary Materials for analyses with the SDO variable).

For the extremity analyses, the Quadratic term entered in Step 2 of the regression was non-significant for the relationships between Political Orientation and Uncertainty-Avoidance, *R*^2^ = 0.009, β = −0.10, *t* = −1.72, *p* = 0.09, bootstrapped *b* = −0.02, 95%BCa CI [−0.04, 0.01] and between Political Orientation and Threat from Unspecified Outgroups, *R*^2^ < 0.001, β = −0.03, *t* = −0.46, *p* = 0.643, bootstrapped *b* = −0.01, 95%BCa CI [−0.04, 0.03] (Hypothesis 2a). In separate regressions, the Quadratic term was also non-significant for the Political Orientation to Attitudes relationship, *R*^2^ = 0.002, β = −0.05, *t* = −0.98, *p* = 0.327, bootstrapped *b* = −0.02, 95%BCa CI [−0.05, 0.02] and also for the Negative Attitudes variable as predicted (Hypothesis 2b), *R*^2^ < 0.001, β = 0.01, *t* = 0.10, *p* = 0.923, bootstrapped *b* < 0.01, 95%BCa CI [−0.05, 0.05]. Next, a binary logistic regression was conducted to test the binary, behavioral-variable of Helping Immigrants who are living in poverty. We again observed that more liberalism was significantly related to more Helping of immigrants (Hypothesis 1a), β = 0.20, *SE* = 0.07, *Wald* = 9.16, *p* = 0.002, *Odds Ratio* = 1.22, 95%BCa CI [1.07, 1.40], Cox and Snell *R*^2^ = 0.03.

#### Mediations

**FIGURE 2:**
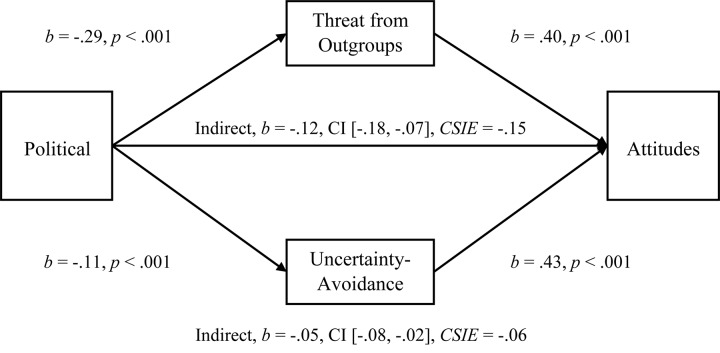
Simultaneous, multiple mediation of political orientation to attitudes toward immigrants with perceived threat from unspecified outgroups and uncertainty-avoidance as mediators. Brackets represent 95% bias corrected confidence intervals from a 5000-sample bootstrap test.

**FIGURE 3:**
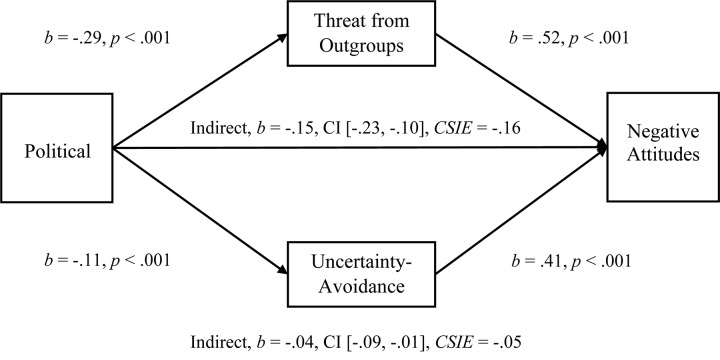
Simultaneous, multiple mediation of political orientation to negative attitudes toward immigrants with perceived threat from unspecified outgroups and uncertainty-avoidance as mediators. Brackets represent 95% bias corrected confidence intervals from a 5000-sample bootstrap test.

To examine the multiple-mediation hypothesis for Perceived Threat from Outgroups and Uncertainty-Avoidance (*r* = 0.157), we conducted separate, multiple-mediation analyses for Attitudes and for Negative Attitudes toward Immigrants using PROCESS. As predicted, both Uncertainty-Avoidance and Perceived Threat from Outgroups had significant indirect effects on the Political Orientation to Attitudes relationship (see Figure 2; Hypothesis 1c), and Threat was larger, comparison *b* = −0.07, CI [−0.14, −0.01]. Both Uncertainty-Avoidance and Threat from Unspecified Outgroups also had significant indirect effects on the Political Orientation to Negative Attitudes relationship (see Figure 3; Hypothesis 1c), and Threat was larger, comparison *b* = −0.11, CI [−0.19, −0.04]. Once again, we tested these two mediations with the Symbolic Threat subscale and replicated the same pattern of results as with the full Perceived Threat scale (see Supplementary Table 1 in Supplementary Materials for full results with Symbolic Threat). The mediational analyses for Behavioral Helping were, however, not supportive (see Supplementary Table 1 in Supplementary Materials for a discussion of issues with placement and reliability).

Confirming the results of Study 1, both mediators were significant when entered into separate mediations for the Political Orientation to Attitudes relationship (Uncertainty-Avoidance, *b* = −0.05, CI [−0.08, −0.02], *R*^2^ = 0.08, *CSIE* = −0.06, and Perceived Threat from Unspecified Outgroups, *b* = −0.12, CI [−0.19, −0.07], *R*^2^ = 0.17, *CSIE* = −0.15]). The separate mediations were also significant for the Political to Negative Attitudes relationship with Uncertainty-Avoidance *b* = −0.05, CI [−0.10, −0.02], *R*^2^ = 0.05, *CSIE* = −0.05, and Perceived Threat from Unspecified Outgroups *b* = −0.15, CI [−0.24, −0.10], *R*^2^ = 0.13, *CSIE* = −0.16.

The Left-Right measure of Political Orientation produced the same pattern of results and levels of significance for all analyses with the exception that the quadratic effect on just the Attitudes measure was now significant; however, this effect was small, *R*^2^ = 0.01, β = −0.21, *t* = −2.11, *p* = 0.036. In addition, for both political variables, all patterns of results and levels of significance remained the same for all analyses in a sample of 301 participants after removing participants who failed to indicate that they were born in the United States in the second set of demographic questions in Study 2. Even though all 308 participants indicated that they were born in the United States in the prolific.ac screening criteria, some had failed to verify this within the Study 2 demographic questions.

### Discussion

In Study 2, we extended the measurement of attitudes to a measure examining only negative responses and also extended the perceived threat from immigrants measure to perceived threat from (unspecified) outgroups in a manner similar to the way SDO is measured in relation to (unspecified) groups. We replicated the political orientation to attitudes results and the multiple-mediations with uncertainty-avoidance and threat that had been observed in Study 1; in this case, we used a more general threat from outgroups instead of threat from immigrants in order to remove common target bias. We also demonstrated the same pattern of results for the Negative Attitudes measure and the Attitudes measure. Overall, the observed results significantly extend the Uncertainty-Threat Model and its implications for examining intergroup relationships, especially for low status or disadvantaged groups such as immigrants.

## Study 3

In Study 3, we replaced the behavioral dependent variable with a validated, implicit measure of attitudes, the Affect Misattribution Procedure (AMP; Payne et al., 2005); we did not measure both variables because of time constraints. In addition, we returned to the use of the perceived threat from immigrants measure as a mediator in Study 3 in order to test whether it would mediate the results to the negative attitudes measure used in Study 2 but not in Study 1; We also tested whether perceived threat would mediate the relationship to the validated, implicit measure.

### Methods

#### Participants

A sample of three hundred and thirty-one participants was recruited from the Prolific.ac to complete the online study with the anticipation that we would have to remove participants for non-completion and inattention. We followed the standard procedure for removing participants who failed to follow instructions and who responded on the implicit bias measure with one key response on ninety-eight percent or more of all test trials (Payne and Lundberg, 2014); we had a final sample of 312 participants. All participants indicated within the Prolific.ac platform that they were born in the US and currently resided in the US; thus, no participant was an immigrant. They were between 18 and 75 years old (*M* = 31.71, *SD* = 12.39) with 68.6% White, 20.8% Conservative, and 54.2% male participants. We recruited roughly 300 participants in order to have 0.8 power for 2 predictors in the model with a small effect size (*d* = 0.38) based upon previous online completion rates for our studies and based upon the results of Studies 1 and 2. See Supplementary Materials for Supplementary Data Sheet 5 (i.e., Data Dictionary for Study 3) and Supplementary Data Sheet 6 (i.e., Data File for Study 3).

#### Materials

Participants completed an online version of the Affect Misattribution Procedure (AMP) as an indirect measure of attitude bias toward immigrants (Payne et al., 2005, 2008, 2010; Imhoff and Banse, 2009; Payne and Lundberg, 2014). In the AMP implicit attitude bias measure, participants saw a photograph of an Immigrant face (Pakistani/Indian face), a Non-Immigrant face (White), or a gray square for an equal number of trials; the prime faces were matched for attractiveness. Each prime photo was replaced quickly with a Korean pictograph of a non-word, letter string; we used non-words so that ability to read Korean did not affect the outcome of the task. We used the same timing as previous online AMP research (Payne et al., 2010) in which there were 72 trials where participants saw a Dot for 500 ms to identify the beginning of a trial and this dot was followed by a prime (face or gray square) for 75 ms, then followed by a pictograph for 225 ms. A television-static pattern mask then appeared and remained on the screen until participants responded by pressing either the key labeled Pleasant or the key labeled Unpleasant. Participants were instructed to ignore the faces of immigrants and non-immigrants and to judge only whether the pictograph drawing appeared to be more or less pleasant than average; they had four practice trials to familiarize themselves with the task prior to completing the 72 critical trials. Thirty-six pictograph, non-words were presented twice within 2 blocks of 36 trials, and the 12 immigrant faces, 12 white faces, and 12 gray squares were presented twice each (i.e., once in each block of 36 trials for a total of 72 trials). Each prime was randomly paired with pictographs within each block of 36 trials. We used the standard scoring procedure from past research by taking the percentage of pleasant responses after White faces and subtracting the percentage of pleasant responses after Immigrant faces; thus, scores can range from 1 to −1 and higher scores indicated more positive attitudes toward White faces in comparison to Immigrant faces (AMP; Payne et al., 2005, 2010).

#### Design

Study 3 used the same materials and procedures as Study 2 with the following changes: (1) In the current study, we returned to the use of the Perceived Threat from Immigrants scale (Stephan et al., 1999) that we had used in Study 1, and (2) In Study 3, we counterbalanced the order of Perceived Threat, Uncertainty-Avoidance, and SDO. The attitude dependent variables were also counterbalanced so that explicit measures and the implicit measure each occurred first for half of the participants in order to control for order effects (see Supplementary Table 1 in Supplementary Materials for the order of measures), (3) we dropped the measure of system threat and the behavioral helping measure, and added the measure of Implicit Attitudes (see Materials for procedural details).

#### Procedure

After completing the demographic questions and the rating of political ideology, participants completed the same Filler Task 1 (four items from the Need for Cognition scale, α = 0.61) used in Studies 1 and 2. Next, participants were randomly assigned to order by being asked to choose the letter that appeared at the top of a list of letters (each randomly ordered for each participant and participants were unaware of the meaning of the letters; see Supplementary Table 1 in Supplementary Materials for orders). For Study 3, Perceived Threat, Uncertainty-Avoidance, and SDO were counterbalanced to control for order effects. After these mediators, participants completed the same Filler Task 2 (α = 0.74) used in Studies 1 and 2, and then completed the attitude dependent variables (Explicit Attitudes, Explicit Negative Attitudes, and Implicit Attitudes), which were counterbalanced so that explicit measures and the implicit measure each occurred first for half of the participants in order to control for order effects. Attitudes toward immigrants, Perceived Threat from Immigrants, and Uncertainty-Avoidance were measured with the same scales used in Study 1 and all three showed good reliability (Attitudes, *M* = 3.60, *SD* = 1.40, α = 0.91; Threat, *M* = 3.00, *SD* = 1.16, α = 0.92; Uncertainty-Avoidance, *M* = 2.93, *SD* = 0.74, α = 0.77) with higher average scores equaling more negative attitudes or more perceived threat. Implicit Attitudes (*M* = 0.00, *SD* = 0.25) were measured with the standard AMP, and Explicit Negative Attitudes (*M* = 2.34, *SD* = 1.68, α = 0.95) were again measured with the same scale used in Study 2. After completing Filler Task 3 (α = 0.64) and two mathematics items, participants were thanked and debriefed.

### Results

We coded the orders of the mediators to categorize Threat first, Uncertainty-Avoidance first, and SDO first orders. We then conducted a regression with Order, Political Orientation, and the interaction in the model for each outcome measure. We observed non-significant Order x Political interactions for each outcome (for the Attitudes toward Immigrants measure, all *p*-values > 0.227 and all *R*^2^ < 0.005; for the Negative Attitude measure, all *p* > 0.230 and all *R*^2^ < 0.001; for the Implicit Attitude measure, all *p* > 0.060 and all *R*^2^ < 0.012). We next conducted three linear regressions to test the expected negative associations between Political Orientation and Explicit Attitudes, Political Orientation and Explicit Negative Attitudes, and Political Orientation and Implicit Attitudes toward Immigrants. Higher scores on Political Orientation indicated a more liberal ideology. As hypothesized, Political Orientation was significantly and negatively related to Explicit Attitudes (Hypothesis 1a), *R*^2^ = 0.23, *β* = −0.48, *t* = −9.58, *p* < 0.001, with bootstrapped *b* = −0.33, 95%BCa CI [−0.41, −0.25], *p* < 0.001, significantly and negatively related to Explicit Negative Attitudes (Hypothesis 1a), *R*^2^ = 0.24, β = −0.49, *t* = −9.99, *p* < 0.001, with bootstrapped *b* = −0.40, 95%BCa CI [−0.50, −0.30], *p* < 0.001, and it also was a significant, negative predictor of Implicit Attitudes (Hypothesis 1a), *R*^2^ = 0.12, β = −0.35, *t* = −6.48, *p* < 0.001, with bootstrapped *b* = −0.04, 95%BCa CI [−0.06, −0.03], *p* < 0.001. We standardized the three dependent variables and averaged them to create a standardized-Averaged Attitude Score. Political Orientation was a significant, negative predictor of standardized-Averaged Attitudes, *R*^2^ = 0.32, β = −0.57, *t* = −12.12, *p* < 0.001, with bootstrapped *b* = −0.21, 95%BCa CI [−0.26, −0.17], *p* < 0.001. It is also of note that participants showed significantly more negative attitudes on the explicit measures (i.e., Attitudes and Negative Attitudes toward Immigrants) than they did on the implicit measure; for example, the change in *R*^2^ was significant when adding Explicit Attitudes to a model predicting Political Orientation from Implicit Attitude Bias, *R*^2^ Change = 0.283, *F*(1, 309) = 60.89, *p* < 0.001. This result is unusual in research on attitudes because social desirability pressures usually encourage people to respond more positively on explicit measures for sensitive topics, while they are less able to alter their responses on the implicit measures; thus, participants usually show less bias on explicit measures.

For the extremity analyses, the Quadratic term that was entered in Step 2 of the regression was non-significant for the relationship between Political Orientation and Uncertainty-Avoidance, β = 0.04, *t* = 0.70, *p* = 0.485, bootstrapped *b* = 0.01, 95%BCa CI [−0.01, 0.03], and Political Orientation and Threat (Hypothesis 2a), β = −0.01, *t* = −0.26, *p* = 0.794, bootstrapped *b* < 0.01, 95%BCa CI [−0.03, 0.03], which again replicated Jost et al. (2003, 2007). In addition, in separate regressions, the Quadratic term was non-significant for Political Orientation relationships with Explicit Attitudes (Hypothesis 2b), *R*^2^ = 0.002, β = −0.04, *t* = −0.78, *p* = 0.434, bootstrapped *b* = −0.01, 95%BCa CI [−0.05, 0.03], with Implicit Attitudes (Hypothesis 2b), *R*^2^ < 0.001, β < 0.001, *t* = 0.21, *p* = 0.834, bootstrapped *b* < 0.01, 95%BCa CI [−0.01, 0.01], and with z-Averaged Attitudes, *R*^2^ < 0.001, β < 0.001, *t* = 0.66, *p* = 0.509, bootstrapped *b* = 0.01, 95%BCa CI [−0.01, 0.03]. It was, however, significant for the Explicit Negative Attitudes variable (Hypothesis 2b), *R*^2^ = 0.01, β = 0.10, *t* = 2.03, *p* = 0.043, bootstrapped *b* = 0.04, 95%BCa CI [−0.01, 0.08], but with a small effect size (*R*^2^ = 0.01 is at the bottom of the small effect size range of *R*^2^ = 0.01 to *R*^2^ = 0.059).

#### Mediations

**FIGURE 4:**
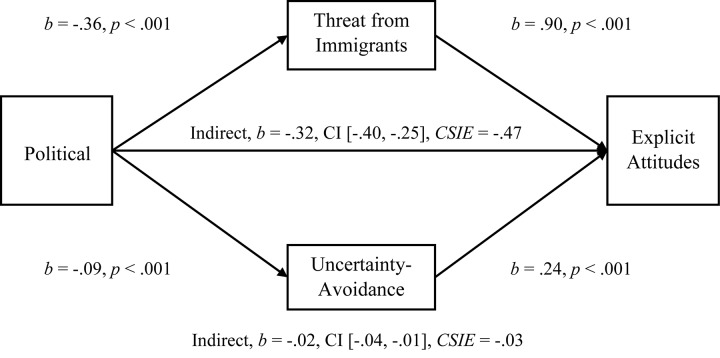
Simultaneous, multiple mediation of political orientation to explicit attitudes toward immigrants with perceived threat from immigrants and uncertainty-avoidance as mediators. Brackets represent 95% bias corrected confidence intervals from a 5000-sample bootstrap test.

**FIGURE 5:**
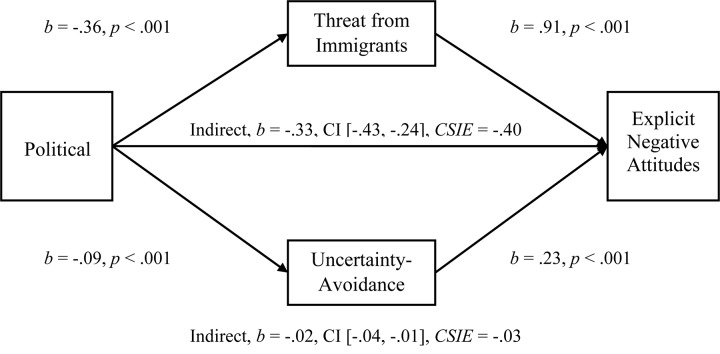
Simultaneous, multiple mediation of political orientation to explicit negative attitudes toward immigrants with perceived threat from immigrants and uncertainty-avoidance as mediators. Brackets represent 95% bias corrected confidence intervals from a 5000-sample bootstrap test.

**FIGURE 6:**
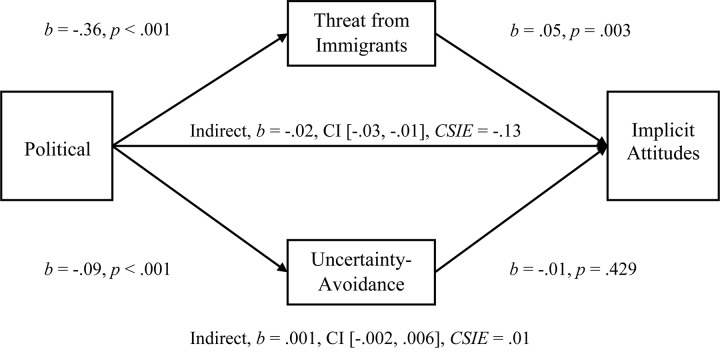
Simultaneous, multiple mediation of political orientation to implicit attitudes with perceived threat from immigrants and uncertainty-avoidance as mediators. Brackets represent 95% bias corrected confidence intervals from a 5000-sample bootstrap test.

**FIGURE 7:**
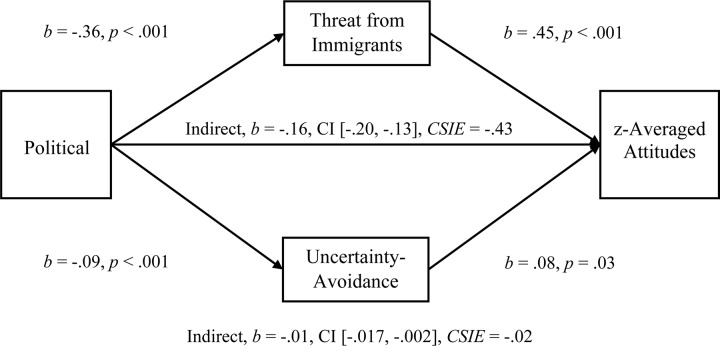
Simultaneous, multiple mediation of political orientation to standardized-averaged attitudes with perceived threat from immigrants and uncertainty-avoidance as mediators. Brackets represent 95% bias corrected confidence intervals from a 5000-sample bootstrap test.

To examine the multiple-mediation hypothesis for Uncertainty-Avoidance and Perceived Threat from Immigrants (*r* = 0.242), we conducted separate, multiple-mediation analyses for the relationships between Political Orientation and Explicit Attitudes, Political Orientation and Explicit Negative Attitudes, Political Orientation and Implicit Attitudes, and Political Orientation and standardized-Averaged Attitudes. As hypothesized, both Uncertainty-Avoidance and Perceived Threat had significant indirect effects on Explicit Attitudes (see Figure 4; Hypothesis 1c), and Threat was a larger mediator, comparison *b* = −0.30, CI [−0.38, −0.23]. In a separate mediation, both Uncertainty-Avoidance and Perceived Threat had significant indirect effects on Negative Attitudes (see Figure 5; Hypothesis 1c), and again Threat was a larger mediator, comparison *b* = −0.31, CI [−0.41, −0.22]. For standardized-Averaged Attitudes, both Uncertainty-Avoidance and Perceived Threat had significant indirect effects on standardized-Averaged Attitudes (see Figure 7). In contrast to expectations, Uncertainty-Avoidance was not a significant mediator when Threat was within the multiple-mediation model for Implicit Attitudes (see Figure 6; Hypothesis 1c).

Both mediators were significant when entered into separate mediations for the relationship between Political Orientation and Explicit Attitudes (Uncertainty-Avoidance, *b* = −0.03, CI [−0.06, −0.02], *R*^2^ = 0.06, *CSIE* = −0.05, and Perceived Threat, *b* = −0.33, CI [−0.41, −0.26], *R*^2^ = 0.23, *CSIE* = −0.48]), and separate mediations for the relationship between Political Orientation and Explicit Negative Attitudes (Uncertainty-Avoidance *b* = −0.03, CI [−0.07, −0.01], *R*^2^ = 0.05, *CSIE* = −0.04, and Perceived Threat *b* = −0.33, CI [−0.44, −0.25], *R*^2^ = 0.24, *CSIE* = −0.41). However, for the relationship between Political Orientation and Implicit Attitudes, Uncertainty-Avoidance was not significant, *b* = 0.0008, CI [−0.0024, 0.0048], *R*^2^ = 0.003, *CSIE* = 0.006, while Perceived Threat was significant *b* = −0.02, CI [−0.03, −0.01], *R*^2^ = 0.09, *CSIE* = −0.13, when entered alone.

The Left-Right measure of Political Orientation produced the same pattern of results and levels of significance for all analyses, except that the Uncertainty-Avoidance indirect effect was not significant within the multiple-mediation including threat on the standardized-Averaged Attitudes measure. After removing an additional four participants who failed to verify that they were born in the US within the Study 3 demographic questions (308 participants), all patterns and levels of significance were the same for the Left-Right measure. Again, for the main political variable, all patterns of results and levels of significance remained the same for all analyses in this sample of 308 participants.

## General Discussion

Our findings support research on the divide between liberals and conservatives by highlighting the importance that differences in threat management and uncertainty-avoidance play in explaining political differences. In three studies, we provided consistent evidence that the Uncertainty-Threat Model predicts relationships of political orientation to threat and uncertainty-avoidance, that it can be extended to an intergroup context of immigration, and that it predicts attitude differences for liberals and conservatives in relation to immigrants. Moreover, both measured *dispositional* variables of uncertainty-avoidance and perceived threat concerns mediated the relationship between more liberalism and less negative attitudes within an intergroup context related to immigrants (Hypothesis 1c). In Study 1, measured uncertainty-avoidance and perceived threats from immigrants mediated the political orientation to attitudes toward immigrants relationship; they did so within both a multiple-mediation model and as separate mediators. Within the multiple-mediation model that included uncertainty-avoidance, the perceived threat mediator was shown to be significantly more influential than uncertainty-avoidance on the relationship. Our research demonstrated the mediation in relation to attitudes toward immigrants, and it also extended threat management beyond the concepts of systemic threat from terrorism, belief in a dangerous world, and death anxiety (Jost et al., 2007). In Study 2, perceived threat was again extended from perceived threat from immigrants to perceived threat from (unspecified) outgroups, and attitudes were extended to include a measure focusing on only negativity. Both uncertainty-avoidance and perceived-threat from (unspecified) outgroups mediated the relationship between more liberalism and less negativity toward immigrants, on both attitude measures. In Study 3, we replicated all findings with the exception of the mediational analysis for the Implicit Attitudes measure. Interestingly, this indirect attitude measure appears to be more tied to threat than we had previously believed because threat was the only significant mediator of the relationship within either mediation model. However, an additional analysis on the averaged attitude measure (average of Explicit Attitudes, Explicit Negative Attitudes, and Implicit Attitudes) revealed that both uncertainty-avoidance and perceived-threat from immigrants significantly mediated the political orientation to averaged attitudes relationship; a combined analysis of Explicit Attitudes and Explicit Negative Attitudes from Study 2 also revealed the same significant mediation for uncertainty-avoidance and general threat.

In our research, we mainly observed evidence supporting the uncertainty-threat model (i.e., linear relationship between political ideology and threat and uncertainty-avoidance [Hypothesis 2a], and between political ideology and attitudes toward immigrants [Hypothesis 2b]), and we did not observe much support for the extremity hypothesis (i.e., a quadratic relationship; van Prooijen et al., 2015). The extremity hypothesis from the worldview perspective proposes that people at either the left or right extreme on the political spectrum should show more extreme reactions than people at the middle of the distribution (Greenberg and Jonas, 2003). Jost et al. (2007) and van Prooijen et al. (2015) used a quadratic regression to test this hypothesis. In accordance with Jost et al.’s findings, we did not observe support for the extremity effect in relation to threat and uncertainty-avoidance in three studies with appropriate sample sizes (206, 308, and 312 participants had 0.8 power to observe an *R*^2^ of 0.025-−0.04) and using a general population and not samples using undergraduate students. In addition, van Prooijen and colleagues demonstrated that people on the extreme left and right showed more derogation of immigrants and more derogation of societal groups in general. In our three studies, we did not find evidence of this extremity hypothesis for 5 of the 6 attitude analyses. We, instead, observed a significant linear effect in 5 analyses in which liberals showed less negative attitudes, but we did not observe the significant quadratic effect; participants at the extreme ends did not show more bias than moderates. We, however, did observe a significant extremity effect on the Negative Attitude measure, but only in Study 3 with a small effect size (*R*^2^ of 0.01), which matches van Prooijen’s effect size of an *R*^2^ of 0.01 (van Prooijen et al., 2015). In their research, they used an extremely large sample of 7,553 participants and observed the same, small change in *R*^2^ of 0.01 (derogation of immigrants) that we observed with 312 participants in our Study 3; the extremity hypothesis effects were all smaller than 0.01 in our other samples of 206 and 308 participants in our Studies 1 and 2. Thus, given that the extremity hypothesis effect size was no larger than an *R*^2^ of 0.01 in samples of 7,553, 206, 308, and 312 participants, it is reasonable to assume that an *R*^2^ of 0.01 might be a good approximation of this effect size in relation to immigrants.

We, however, did not include a measure of derogation to societal groups as van Prooijen and colleagues did and for which they observed a larger effect size (*R*^2^ = 0.03). So, using other groups may be related to a larger effect. Yet, it may also be related to methodology because they tested attitudes to multiple groups simultaneously, instead of individually, which could have changed responses compared to rating a single group such as immigrants. Question order effects have been observed in social cognition research in which an earlier question frames the questions that follow; thus, the use of multiple groups could affect the responses (Schwarz et al., 1990; Schuldt et al., 2015). This is an empirical question for future studies to tackle.

We also analyzed the type of threat we used within our research. A recent review has indicated that threat has been defined within the motivated social cognition perspective (Jost et al., 2003; Jost, 2017) in a way that may miss differences (Crawford, 2017). This review suggests that *physical threat* (i.e., more concrete concerns regarding violation of physical safety through the potential of death or physical harm) and *meaning threats* (i.e., more abstract threats to systems of meaning and values) have often been conflated. They argue that liberals and conservatives respond differently to physical threats, but that they usually respond in a similar manner to meaning threats. Within our current research, we did not focus on *physical threats*. Instead we focused on perceived threats based upon the ITT perspective (Stephan et al., 2009, 2015; Rios et al., 2018) that has not distinguished between perceived realistic threats about competition for resources (e.g., taking jobs; using social services) and perceived realistic threats about physical safety (e.g., threats of death or physical trauma). The ITT theory has instead focused on perceived threats to symbolic values and threats to resources of one’s groups. Thus, the standard perceived threat measure that we used (Stephan et al., 1999, 2009) assessed perceived threats to resources and perceived threats to symbolic values, but to date, has not measured threats from the potential of death or other physical harm even though it could; As a result, the measure of perceived threats that we used was closer to measuring *meaning threats.* Contrary to the review of *meaning threats*, we observed that liberals and conservatives responded differently to perceived threats (i.e., combined perceived realistic and symbolic threat similar to meaning threats), and even the symbolic threat subscale entirely replicated the differential relationship to political ideology and the mediations to implicit and explicit attitudes toward immigrants.

In relation to uncertainty, we had used personality measures of uncertainty-avoidance that had been used previously (Jost et al., 2007; Jost and Napier, 2012). Over our three studies, we observed a weighted-average beta between Political Ideology and Uncertainty-Avoidance of 0.25 (*R*^2^ = 0.06); while this was a moderate effect size and was the reason we kept it throughout our three studies, the weighted-average beta observed by Jost et al. (2007) was higher (0.41). We have reviewed the recent research by Jost (2017) and Jost et al. (2017) and have noted that Uncertainty-Avoidance may have a larger correlation with Political Ideology when more of the items of Need for Closure or Need for Structure are included within the measures of uncertainty-avoidance. Thus, future research may improve the measurement of Uncertainty-Avoidance by including Need for Cognitive Closure within this measure.

The current research provides a good initial test of the chronic dispositional hypotheses that can be derived from the Uncertainty-Threat Model, but there are some limitations. Given that this research was an initial test, it was correlational and limited to only demonstrating associations for the Uncertainty-Threat Model and its relationships to attitudes toward immigrants, though we did use methods to improve the correlational studies. For the mediational analyses, by measuring the mediators after political ideology, and attitudes after the mediators, it is known that the mediators did not influence participants’ ratings of political ideology within the study because the political ratings occurred first, and that attitudes did not influence participants’ ratings of the mediators or political ideology (Kenny et al., 1998; Cohen et al., 2003). However, rating political ideology could have had an influence on the later measures, though this a common issue with political studies. Thus, future research could alter the order of these variables to fully demonstrate that those orders did not influence ratings. Moreover, future research could experimentally test the hypotheses generated by this model in order to examine the Uncertainty-Threat Model’s proposal that temporarily activating uncertainty and threat may induce a conservative shift in addition to the effects observed with chronic, dispositional measures. This is a question that the current research is not able to answer. Another limitation is the nature of the perceived threat that we measured. According to ITT, perceived realistic threats can be about both competition for resources (e.g., taking jobs; using social services) and about physical safety (e.g., being physically hurt). The standard measures used for measuring perceived threat based upon ITT have not measured threats to physical safety at the concrete or conceptual level, but according to the theory, there is room for incorporating such items (Stephan et al., 1999, 2002). If those physical safety items were to be included, we may see differences between the realistic and perceived threat subscales, which is often not observed on the usual measures (Rios et al., 2018). Thus, more research on the nature of this measure will need to be conducted to test how well it taps meaning threats as opposed to physical threats, and whether physical threats can be measured more broadly and less concretely.

In addition to showing support for the Uncertainty-Threat Model, this research extended the model to perceived threat from outgroups, both to specific outgroups (i.e., immigrants) and to unspecified outgroups much like SDO does with unspecified groups, and it extended threat beyond systemic threat from terrorism, belief in a dangerous world, or death anxiety (Jost et al., 2007). In Study 1, we used a validated measure of perceived threat from immigrants and found a significant mediation of the relationship between more liberalism and less negative attitudes toward immigrants (Stephan et al., 1999). In Study 2, we extended this research by testing the model with a measure of perceived threat from unspecified outgroups and observed a similar significant mediation; moreover, the use of unspecified groups in the threat measure, but “immigrants” in the attitude measures removed common target bias effects and improved generalizability. In Study 3, we returned to the measure of perceived threat from immigrants and demonstrated its mediation of the political orientation to attitudes effect for an objective, indirect measure of attitudes. Moreover, in all three studies, perceived threat was a significantly more influential mediator than uncertainty-avoidance within the multiple-mediation model. This effect highlights the importance that perceived threat may play in differentiating liberals and conservatives and may provide a topic to bridge the divide on the immigration debates currently occurring worldwide.

## Conclusion and Implications

A key difference between liberals and conservatives may be their responsiveness to perceived threats or risks and uncertainty (Jost et al., 2017) instead of differences in negativity bias as proposed by some theorists (Hibbing et al., 2014). Other researchers have begun to show that liberals and conservatives may differ in relation to threats (Oxley et al., 2008; Thórisdóttir and Jost, 2011; Terrizzi et al., 2013). In other recent research, conservatism was positively related to self-restraint and social-order dimensions of the Moral Motives Model (Janoff-Bulman et al., 2007), both of which could be said to be related to differences in responsiveness to social threats. Discussing these differences in terms of people having different responsiveness to threat, as opposed to wanting to be socially dominant or having a general negativity bias, allows for a constructive debate that may reduce the focus of casting either group in a negative light. Differences in responsiveness to threat can be adaptive in different circumstances and having a balance of both may be beneficial to society (Janoff-Bulman and Carnes, 2016). If we are able to acknowledge the perceived threats, it allows for the discussion of the benefits of immigration to the overall economy, while also discussing the problems with immigration hurting the poor with whom immigrants occasionally compete for jobs. It allows us to focus on helping immigrants learn new, societal systems and to improve the shared focus on the ideals of the principles of democracy rather than on dividing us on cultural differences defined by blood ties and heritage. It permits these groups to discuss issues related to threat in ways that may acknowledge and offer solutions to differences in threat and perhaps move the debate forward and toward the promise of democracy instead of further dividing us.

## Ethics Statement

This study was carried out in accordance with the recommendations of the University Code of Practice for Research, and the Science, Technology, Engineering and Mathematics Ethical Review Committee. The protocol was approved by the Science, Technology, Engineering and Mathematics Ethical Review Committee at the University of Birmingham. All subjects gave written informed consent in accordance with the Declaration of Helsinki.

## Author Contributions

BS conceived the original study idea with FG and DM providing significant input in its development and the general direction and development of the line of research. BS, FG, and DM developed the study materials. Study design and analysis of data was conducted by FG and DM with assistance by BS. Writing up of the studies was done by BS and FG, with assistance and revision from DM.

## Conflict of Interest Statement

The authors declare that the research was conducted in the absence of any commercial or financial relationships that could be construed as a potential conflict of interest.
