# Supplementary material for: Bridging Political Divides: Perceived Threat and Uncertainty Avoidance Help Explain the Relationship Between Political Ideology and Immigrant Attitudes Within Diverse Intergroup Contexts
Source: Front Psychol. 2019 Jun 14;10:1236. doi: 10.3389/fpsyg.2019.01236 (PMC6587119; doi:10.3389/fpsyg.2019.01236)
Supplement: Supplementary file 1 [file Table_1.docx]

**TABLE 1 of the SUPPLEMENTAL MATERIALS**

**Study 2:** Perceived Threat from Outgroups (adapted from Stephan, Boniecki, Ybarra, Bettencourt, Ervin, Jackson, McNatt, & Renfro, 2002).

**Instructions**: Please take a moment now to complete the following questions.

This is a study of attitudes toward outgroups. Specifically, the purpose of this study is to understand the views toward people not from your ingroup. We are asking questions about some sensitive issues. We recognize that sometimes it is not easy to be completely frank and honest, but your answers will help us better understand attitudes toward these important issues.

Obviously, there are no right or wrong answers to any of these questions. All that matters is your own opinion on these issues. Your responses are completely confidential. There is no identifying information on this questionnaire that would link the responses to you.

In this section of the study, we will ask you to think about "OUTGROUPS." For the purposes of this study, an Outgroup is any group or groups of which you DO NOT class yourself as being a member of, or belonging to, and that you do not identify with.

(click next to begin)

1. Outgroups hold too many positions of power and responsibility in this country.

o Disagree Strongly

o Disagree Moderately

o Disagree Somewhat

o Neither Agree nor Disagree

o Agree Somewhat

o Agree Moderately

o Agree Strongly

2. Outgroups dominate American politics more than they should.

3. When outgroups are in positions of authority, they discriminate against my group when making hiring decisions.

4. Too much money is spent on educational programs that benefit outgroups.

5. Outgroups have more economic power than they deserve in this country.

6. Outgroups receive too much of the money spent on healthcare and childcare.

7. Too little money per student is spent on education for outgroups.

8. The tax system favors outgroups.

9. Many companies hire less qualified members of outgroups over more qualified members of my group.

10. Outgroups have more political power than they deserve in this country.

11. Public service agencies favor outgroups over my group.

12. The legal system is stricter on outgroups than on my group.

13. My group has very different values than outgroups.

14. Outgroups have no right to think they have better values than my group.

15. Outgroups want their rights to be put ahead of the rights of my group.

16. Outgroups don’t understand the way my group views the world.

17. Outgroups do not value the rights granted by the Constitution (life,

liberty, and the pursuit of happiness) as much as my group does.

18. Outgroups and my group have different family values.

19. Outgroups don’t value the traditions of their group as much as my group does.

20. Outgroups regard themselves as morally superior to my group.

21. The values of outgroups regarding work are different from those of my group.

22. Most members of outgroups will never understand what members of my group are like.

23. Outgroups should not try to impose their values on my group.

24. My group does not get as much respect from outgroups as they deserve.

**Counterbalancing**

**Study 1:**  Counterbalancing the order of measures.

| **Order** | **Measures** | | | | | | |
| --- | --- | --- | --- | --- | --- | --- | --- |
|  | Mediators | | | Filler Task 2 | Outcomes | Secondary measures |  |
| 1 | Threat | UA: Openness1st | UA: Ambiguity2nd | Ncog2nd4 | Attitudes | Death Avoid, Fear of Death, System Threat | SDO |
| 2 | Threat | UA: Ambiguity1st | UA: Openness2nd | Ncog2nd4 | Attitudes | Death Avoid, Fear of Death, System Threat | SDO |
| 3 | UA: Openness1st | UA: Ambiguity2nd | Threat | Ncog2nd4 | Attitudes | Death Avoid, Fear of Death, System Threat | SDO |
| 4 | UA: Ambiguity1st | UA: Openness2nd | Threat | Ncog2nd4 | Attitudes | Death Avoid, Fear of Death, System Threat | SDO |

**Study 2:** Counterbalancing the order of measures.

| **Orders** | **Measures** | | | | |
| --- | --- | --- | --- | --- | --- |
|  | Mediators | | Filler Task 1 | Outcome Measures |  |
| 1 | Threat | U-A | Need for Cognition4 | Explicit Attitudes & Negative Attitudes | SDO |
| 2 | U-A | Threat | Need for Cognition4 | Explicit Attitudes & Negative Attitudes | SDO |

**Study 3:**  Counterbalancing the order of measures.

| **Order** | **Measures** | | | | | | |
| --- | --- | --- | --- | --- | --- | --- | --- |
|  | Mediators | | | Filler Task 1 | Outcomes | Filler2 | Outcomes |
| 1 | Threat | U-A | SDO | N. Cog4 | Explicit Attitudes & Neg Att. | N. Cog | Implicit Attitudes |
| 2 | Threat | SDO | U-A | N. Cog4 | Explicit Attitudes & Neg Att. | N. Cog | Implicit Attitudes |
| 3 | U-A | Threat | SDO | N. Cog4 | Explicit Attitudes & Neg Att. | N. Cog | Implicit Attitudes |
| 4 | U-A | SDO | Threat | N. Cog4 | Explicit Attitudes & Neg Att. | N. Cog | Implicit Attitudes |
| 5 | SDO | Threat | U-A | N. Cog4 | Explicit Attitudes & Neg Att. | N. Cog | Implicit Attitudes |
| 6 | SDO | U-A | Threat | N. Cog4 | Explicit Attitudes & Neg Att. | N. Cog | Implicit Attitudes |
| 7 | Threat | U-A | SDO | N. Cog4 | Implicit Attitudes | N. Cog | Explicit Attitudes & Neg Att. |
| 8 | Threat | SDO | U-A | N. Cog4 | Implicit Attitudes | N. Cog | Explicit Attitudes & Neg Att. |
| 9 | U-A | Threat | SDO | N. Cog4 | Implicit Attitudes | N. Cog | Explicit Attitudes & Neg Att. |
| 10 | U-A | SDO | Threat | N. Cog4 | Implicit Attitudes | N. Cog | Explicit Attitudes & Neg Att. |
| 11 | SDO | Threat | U-A | N. Cog4 | Implicit Attitudes | N. Cog | Explicit Attitudes & Neg Att. |
| 12 | SDO | U-A | Threat | N. Cog4 | Implicit Attitudes | N. Cog | Explicit Attitudes & Neg Att. |

**Materials and Analyses with Social Dominance Orientation (SDO)**

*Social Dominance Orientation scale (SDO):* The Social Dominance Orientation measure included 16 items (Pratto et al., 1994) such as “To get ahead in life, it is sometimes necessary to step on other groups.” All items were completed on a 7-point Likert scale ranging from (1) *Very Negative* to (7) *Very Positive*. Items were presented in a random order. After reverse scoring eight items, the items were averaged with higher scores reflecting higher social dominance ideology (*M* = 2.58, *SD* = 1.25, α = .95).

In order to provide an additional test for our mediational hypotheses, we wished to include another variable within the model that is known to be associated with attitudes and outgroup prejudice. A large amount of research has demonstrated that social dominance orientation (SDO) is associated with negative attitudes toward outgroups (Cohrs & Asbrock, 2009; Duckitt & Sibley, 2010b; Guimond, Dambrun, Michinov, & Durate, 2003; Kteily, Sidanius, & Levin, 2011). Those high in SDO endorse beliefs in a competitive society and a preference for hierarchy within society and for their group to be at the top. This perception along with perceived threats can both be related to increased negative attitudes toward outgroups; thus, we wished to test that Perceived Threat and Uncertainty-Avoidance would remain significant when SDO was added (Hypothesis 3a).

Hypothesis 3a: Perceived-Threat and Uncertainty-Avoidance would remain significant when Social Dominance Orientation (SDO) was added the mediation model for Political Ideology to Attitudes toward Immigrants.

**Study 1:**

We first replicated past research in demonstrating that higher liberalism was significantly related to less SDO, *R^2^* = .29, *β* = -.54, *t* = -9.04, *p* < .001, with bootstrapped *b* = -.31, 95%BCa CI [-.38, -.24], *p* < .001. In addition, both Uncertainty-Avoidance (*b* = -.02, CI [-.05, -.01], *Completely Standardized Indirect Effect (CSIE)* = -.03) and Threat (*b* = -.32, CI [-.41, -.24], *CSIE* = -.45) remained significant indirect effects when SDO (*b* = .01, CI [-.04, .05], *CSIE* = .01) was added to the model (Hypothesis 3a).

**Study 2**:

The SDO finding from Study 1 was also replicated with a similar result showing that higher liberalism was significantly related to less SDO, *R^2^* = .22, *β* = -.47, *t* = -9.41, *p* < .001, with bootstrapped *b* = -.27, 95%BCa CI [-.33, -.21], *p* < .001. Both Uncertainty-Avoidance, *b* = -.03, CI [-.06, -.01], *CSIE* = -.04, and general Threat from Outgroups, *b* = -.08, CI [-.15, -.03] *CSIE* = -.10 on Attitudes toward immigrants, and Uncertainty-Avoidance, *b* = -.03, CI [-.07, -.01], *CSIE* = -.03, and general Threat from Outgroups, *b* = -.11, CI [-.19, -.06], *CSIE* = -.11 on Negative Attitude remained significant indirect effects when SDO was added to the model for both Attitudes and Negative Attitudes respectively (Hypothesis 3a).

**Study 3**:

The SDO findings from Studies 1 and 2 were also replicated with similar results showing that higher liberalism was significantly related to less SDO, *R^2^* = .31, *β* = -.56, *t* = -11.74, *p* < .001, with bootstrapped *b* = -.29, 95%BCa CI [-.35, -.23], *p* < .001. Both Uncertainty-Avoidance, *b* = -.02, CI [-.04, -.01], *CSIE* = -.03, *b* = -.02, CI [-.039, -.003], *CSIE* = -.02, and *b* = -.01, CI [-.0153, -.0003], *CSIE* = -.02, and Threat, *b* = -.28, CI [-.36, -.21], *CSIE* = -.41, *b* = -.26, CI [-.36, -.18], *CSIE* = -.32, and *b* = -.14, CI [-.19, -.11], *CSIE* = -.38, remained significant indirect effects when SDO was added to the model for both Attitudes toward immigrants , Negative Attitudes, and standardized-Averaged Attitudes respectively (Hypothesis 3a). However, neither Uncertainty-Avoidance *b* = .001, CI [-.002, .005], nor SDO *b* = .004, CI [-.006, .014] were significant indirect effects with Threat included within the multiple-mediation for Implicit Attitudes.

The Left-Right measure of Political Orientation produced the same pattern of results and significances for all analyses, except that Uncertainty-Avoidance was also not significant in the multiple-mediation model with SDO and Threat on the standardized-Averaged Attitudes measure. After removing an additional four participants who failed to verify that they were born in the US within the Study 3 demographic questions (308 participants), all patterns and significances were the same for the Left-Right measure with the exception that Uncertainty-Avoidance was not significant in the multiple mediation with Threat and SDO on the standardized-Averaged Attitudes measure.

**Materials and Analyses of the Secondary Measures**

*Secondary measures*:

*Death Avoidance scale*: This scale consisted of 5 items that were rated on 7-point scales from (1) *Disagree Strongly* to (7) *Agree Strongly*. An example item includes “I avoid death thoughts at all costs.” Higher scores represented more death avoidance (*M* = 3.73, *SD* = 1.68, *α* = .92; Wong, Reker, & Gesser, 1994).

*Fear of Death scale*: This scale consisted of 7 items that were rated on 7-point scales from (1) *Disagree Strongly* to (7) *Agree Strongly*. An example item includes “I have an intense fear of death.” Higher scores represented more fear of death (*M* = 3.91, *SD* = 1.58, *α* = .91; Wong, Reker, & Gesser, 1994).

*System Threat item*: Participants completed a single item on system threat that was used by Jost et al., 2007, “Our way of life is seriously threatened by the forces of terrorism in the world.” This item was rated on 7-point scale from (1) *Disagree Strongly* to (7) *Agree Strongly*. Higher scores represented more system threat.

**Study 1:**

Higher liberalism was significantly related to less System Threat (Hypothesis 3b), *R^2^* = .09, *β* = -.31, *t* = -4.58, *p* < .001, with bootstrapped *b* = -.25, 95%BCa CI [-.36, -.15], *p* < .001. However, we did not replicate Jost et al.’s (2007) findings of Political Orientation predicting Death Avoidance (Hypothesis 3c), *R^2^* = .01, *β* = -.11, *t* = -1.60 *p* = .111, with bootstrapped *b* = -.09, 95%BCa CI [-.19, .02], *p* = .101, or Fear of Death (Hypothesis 3d), *R^2^* < .01, *β* = .05, *t* = 0.76, *p* = .448, with bootstrapped *b* = .04, 95%BCa CI [-.06, .14], *p* = .444.

**Study 2**:

Death Avoidance and Fear of Death were not included in Study 2, but System Threat was. Higher liberalism was significantly related to less System Threat (Hypothesis 3b), *R^2^* = .19, *β* = -.43, *t* = -8.42, *p* < .001, with bootstrapped *b* = -.42, 95%BCa CI [-.50, -.33], *p* < .001.

**Mediations with Perceived Symbolic Threat**

Some researchers have suggested that differences on political ideology and threat are observed mainly on measures of threat related to physical threats such as threats of death or bodily harm (Burke et al., 2013; Crawford, 2017). They suggest that for meaning threats, “threats to systems of meaning and value,” liberals and conservatives should show a symmetry in their responsiveness to these meaning threats. However, if we review the Perceived Threat measure (Stephan et al., 1999), we see that no item within either the Symbolic or Realistic subscales mentions the ideas of “threats of death or bodily harm,” nor do they mention terrorism or physical safety such as committing crimes. Thus, the Perceived Threat measure that we have employed does not measure “*Physical Threats”*. However, to provide peace of mind, we can analyze the Symbolic Threat subscale separately to show that the Symbolic Threat scale replicates the pattern of data of the entire Perceived Threat scale

**Study 1:**

For the Political Orientation to Attitudes toward Immigrants relationship, both Uncertainty-Avoidance (*b* = -.03, CI [-.06, -.01], *CSIE* = -.04) and Symbolic (meaning) Threat (*b* = -.26, CI [-.34, -.19], *CSIE* = -.36) were significant mediators (Figure S1).

**Figure S1**

Political

Symbolic Threat

*b* = -.34, *p* < .001

Attitudes

Uncertainty-Avoidance

*b* = -.07, *p* = .005

*b* = .75, *p* < .001

*b* = .39, *p* < .001

Indirect, *b* = -.26, CI [-.34, -.19], *CSIE* = -.36

Indirect, *b* = -.03, CI [-.06, -.01], *CSIE* = -.04

**Figure S1.** Multiple mediation of Political Orientation to Attitudes toward Immigrants relationship with Symbolic Threat and Uncertainty-Avoidance as mediators. Brackets represent 95% bias corrected confidence intervals from a 5000-sample bootstrap test.

**Study 2:**

For the Political Orientation to Attitudes toward Immigrants relationship, both Uncertainty-Avoidance (*b* = -.05, CI [-.08, -.02], *CSIE* = -.06) and Generalized Symbolic (meaning) Threat (*b* = -.11, CI [-.17, -.06], *CSIE* = -.13) were significant mediators (Figure S2a). For the Political Orientation to Negative Attitudes relationship, both Uncertainty-Avoidance (*b* = -.05, CI [-.09, -.02], *CSIE* = -.05) and Generalized Symbolic (meaning) Threat (*b* = -.14, CI [-.21, -.09], *CSIE* = -.15) were significant mediators (Figure S2b).

**Figure S2a**

Political

Generalized Symbolic Threat

*b* = -.28, *p* < .001

Attitudes

Uncertainty-Avoidance

*b* = -.11, *p* < .001

*b* = .39, *p* < .001

*b* = .44, *p* < .001

Indirect, *b* = -.11, CI [-.17, -.06], *CSIE* = -.13

Indirect, *b* = -.05, CI [-.08, -.02], *CSIE* = -.06

**Figure S2a.** Multiple mediation of Political Orientation to Attitudes toward Immigrants relationship with Generalized Symbolic Threat and Uncertainty-Avoidance as mediators. Brackets represent 95% bias corrected confidence intervals from a 5000-sample bootstrap.

**Figure S2b**

Political

Generalized Symbolic Threat

*b* = -.28, *p* < .001

Negative Attitudes

Uncertainty-Avoidance

*b* = -.11, *p* < .001

*b* = .51, *p* < .001

*b* = .43, *p* < .001

Indirect, *b* = -.14, CI [-.21, -.09], *CSIE* = -.15

Indirect, *b* = -.05, CI [-.09, -.02], *CSIE* = -.05

**Figure S2b.** Multiple mediation of Political Orientation to Negative Attitudes with Generalized Symbolic Threat and Uncertainty-Avoidance as mediators. Brackets represent 95% bias corrected confidence intervals from a 5000-sample bootstrap.

**Study 3:**

For the Political Orientation to Attitudes toward Immigrants relationship, both Uncertainty-Avoidance (*b* = -.02, CI [-.04, -.01], *CSIE* = -.03) and Symbolic Threat (*b* = -.25, CI [-.32, -.19], *CSIE* = -.37) were significant mediators (Figure S3a). For the Political Orientation to Negative Attitudes relationship, both Uncertainty-Avoidance (*b* = -.02, CI [-.04, -.01], *CSIE* = -.03) and Symbolic Threat (*b* = -.25, CI [-.34, -.18], *CSIE* = -.31) were significant mediators (Figure S3b). Finally, for the Political Orientation to Implicit Attitudes relationship, Uncertainty-Avoidance was non-significant (*b* = .001, CI [-.002, .006], *CSIE* = .01) while Symbolic Threat (*b* = -.01, CI [-.02, -.01], *CSIE* = -.10) was significant (Figure S3c).

**Figure S3a**

Political

Symbolic Threat

*b* = -.34, *p* < .001

Attitudes

Uncertainty-Avoidance

*b* = -.09, *p* = .005

*b* = .74, *p* < .001

*b* = .24, *p* = .002

Indirect, *b* = -.25, CI [-.32, -.19], *CSIE* = -.37

Indirect, *b* = -.02, CI [-.04, -.01], *CSIE* = -.03

**Figure S3a.** Multiple mediation of Political Orientation to Attitude s toward Immigrants relationship with Symbolic Threat and Uncertainty-Avoidance as mediators. Brackets represent 95% bias corrected confidence intervals from a 5000-sample bootstrap test.

**Figure S3b**

Political

Symbolic Threat

*b* = -.34, *p* < .001

Negative Attitudes

Uncertainty-Avoidance

*b* = -.09, *p* < .001

*b* = .75, *p* < .001

*b* = .23, *p* = .019

Indirect, *b* = -.25, CI [-.34, -.18], *CSIE* = -.31

Indirect, *b* = -.02, CI [-.04, -.01], *CSIE* = -.03

**Figure S3b.** Multiple mediation of Political Orientation to Negative Attitudes with Symbolic Threat and Uncertainty-Avoidance as mediators. Brackets represent 95% bias corrected confidence intervals from a 5000-sample bootstrap.

**Figure S3c**

Political

Symbolic Threat

*b* = -.34, *p* < .001

Implicit Attitudes

Uncertainty-Avoidance

*b* = -.09, *p* < .001

*b* = .04, *p* = .006

*b* = -.02, *p* = .429

Indirect, *b* = -.01, CI [-.02, -.01], *CSIE* = -.10

Indirect, *b* = .001, CI [-.002, .006], *CSIE* = .01

**Figure S3c.** Multiple mediation of Political Orientation to Implicit Attitudes with Symbolic Threat and Uncertainty-Avoidance as mediators. Brackets represent 95% bias corrected confidence intervals from a 5000-sample bootstrap.

**Mediations with Behavioral Helping variable**

**Study 1:**

For the Political Orientation to Behavioral Helping relationship, Uncertainty-Avoidance was non-significant (*b* = .004, CI [-.025, .041]) while Threat (*b* = .19, CI [.06, .32]) was a significant mediator (Figure b1).

**Figure b1**

Political

Threat

*b* = -.35, *p* < .001

Behavioral Helping

Uncertainty-Avoidance

*b* = -.07, *p* = .005

*b* = -.53, *p* = .005

*b* = -.06, *p* = .782

Indirect, *b* = .19, CI [.06, .32]

Indirect, *b* = .004, CI [-.025, .041]

**Figure b1.** Multiple mediation of Political Orientation to Behavioral Helping with Threat and Uncertainty-Avoidance as mediators. Brackets represent 95% bias corrected confidence intervals from a 5000-sample bootstrap test.

**Study 2:**

For the Political Orientation to Behavioral Helping relationship, neither Uncertainty-Avoidance (*b* = .01, CI [-.03, .05]) nor Threat from Outgroups (*b* = -.03, CI [-.09, .04]) were significant mediators (Figure b2).

**Figure b2**

Political

Threat from Outgroups

*b* = -.29, *p* < .001

Behavioral Helping

Uncertainty-Avoidance

*b* = -.11, *p* < .001

*b* = .09, *p* = .392

*b* = -.12, *p* = .489

Indirect, *b* = -.03, CI [-.09, .04]

Indirect, *b* = .01, CI [-.03, .05]

**Figure b2.** Multiple mediation of Political Orientation to Behavioral Helping with Threat from Outgroups and Uncertainty-Avoidance as mediators.

**References for the Supplemental Materials**

Cohrs, J. C., & Asbrock, F. (2009). Right-wing authoritarianism, social dominance orientation, and prejudice against threatening and competitive ethnic groups? *European Journal of Social Psychology*, *39*, 270–289.

Duckitt, J., & Sibley, C. G. (2010b). Right-Wing Authoritarianism and Social Dominance Orientation differentially moderate intergroup effects on prejudice. *European Journal of* *Personality*, *24*, 583–601.

Guimond, S., Dambrun, M., Michinov, N., & Duarte, S. (2003). Does social dominance generate prejudice? Integrating individual and contextual determinants of intergroup cognitions. *Journal of Personality and Social Psychology*, *84*, 697–721.

Kteily, N. S., Sidanius, J., & Levin, S. (2011). Social Dominance Orientation: Cause or “mere effect”? Evidence for SDO as a causal predictor of prejudice and discrimination against ethnic and racial outgroups. *Journal of Experimental Social Psychology*, *47*, 208–214.
